# MIF contribution to progressive brain diseases

**DOI:** 10.1186/s12974-023-02993-6

**Published:** 2024-01-04

**Authors:** Agata Matejuk, Gil Benedek, Richard Bucala, Szymon Matejuk, Halina Offner, Arthur A. Vandenbark

**Affiliations:** 1 Department of Immunology, Collegium Medicum, University of Zielona Góra, Zielona Góra, Poland; 2 Tissue Typing and Immunogenetics Unit, Department of Genetics, Hadassah Medical Organization and Faculty of Medicine, Hebrew University of Jerusalem, Jerusalem, Israel; 3 Department of Internal Medicine, Section of Rheumatology, Allergy and Immunology, Yale University School of Medicine, New Haven, CT 06520 USA; 4 Jagiellonian University, Cracow, Poland; 5 Neuroimmunology Research, R&D-31, VA Portland Health Care System, 3710 SW U.S. Veterans Hospital Rd., Portland, OR 97239 USA; 6 Department of Neurology, Oregon Health and Science University, 3181 SW Sam Jackson Park Rd, Portland, OR 97239 USA; 7 Department of Anesthesiology and Perioperative Medicine, Oregon Health & Science University, 3181 SW Sam Jackson Park Rd, Portland, OR 97239 USA; 8 Department of Molecular Microbiology and Immunology, Oregon Health & Science University, 3181 SW Sam Jackson Park Rd, Portland, OR 97239 USA

**Keywords:** MIF, Neuroinflammation, Neurodegeneration, MS, AD, Astrocytoma

## Abstract

Progressive brain diseases create a huge social and economic burden on modern societies as a major cause of disability and death. Incidence of brain diseases has a significantly increasing trend and merits new therapeutic strategies. At the base of many progressive brain malfunctions is a process of unresolved, chronic inflammation. Macrophage migration inhibitory factor, MIF, is an inflammatory mediator that recently gained interest of neuro-researchers due to its varied effects on the CNS such as participation of nervous system development, neuroendocrine functions, and modulation of neuroinflammation. MIF appears to be a candidate as a new biomarker and target of novel therapeutics against numerous neurologic diseases ranging from cancer, autoimmune diseases, vascular diseases, neurodegenerative pathology to psychiatric disorders. In this review, we will focus on MIF’s crucial role in neurological diseases such as multiple sclerosis (MS), Alzheimer’s disease (AD) and glioblastoma (GBM).

## Introduction

**Fig. 1:**
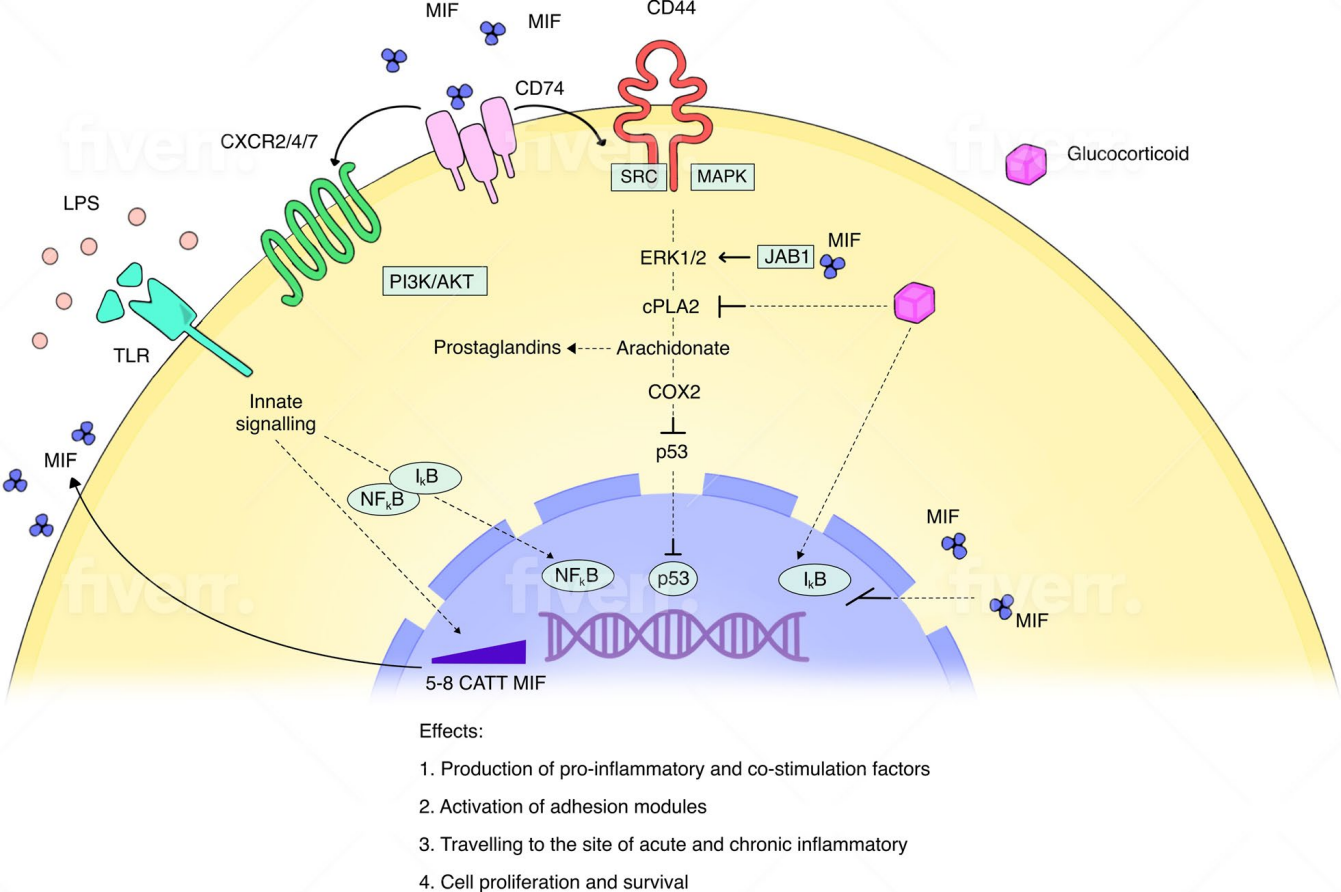
MIF function and signaling. MIF fulfills its biological functions through membrane receptors and via binding to intracellular molecules. MIF’s binding to membrane receptor CD74 recruits CD44 and leads to activation of Src/MAPK signaling. MIF via CXCR2/4 activates PI3K/Akt downstream signaling and induces cell migration. Sustained activation of ERK1/2 phosphorylation is mediated by JUN activation domain binding protein-1 (JAB1) and leads to cytoplasmic phospholipase A2 (cPLA2) activity (blocked by glucocorticoids) and further to arachidonate/prostaglandin production. MIF production can be stimulated via TLRs by e.g., LPS stimulation. MIF regulates innate immune responses through modulation of TLR4. In response to LPS and Gram-negative bacteria MIF upregulates TLR-4 expression and in consequence induces the production of pro-inflammatory cytokines. MIF overrules glucocorticoid effects such as the nuclear factor-κB (NF-κB) inhibitor IκB which downregulates inflammatory responses. MIF via p53 inhibits activation-induced apoptosis, increase cellular survival and proliferation. MIF’s functions include: 1. stimulation of proinflammatory and co-stimulatory factors; 2. activation of adhesion molecules; 3. increase of cell trafficking to the sites of inflammation; 4. increase of cell proliferation and survival and inhibition of apoptosis

During immune responses, a galaxy of factors are activated to maintain homeostasis. Disease occurs when the homeostasis is broken, and inflammation persists. One of the inflammatory mediators with broad influence on host immune responses is MIF. MIF, a cytokine, hormone and enzyme, was first described almost a half century ago in association with delayed-type hypersensitivity [1, 2]. At that time, Bloom and Bennett in an in vitro study showed the inhibition of macrophage migration mediated by a soluble factor produced by sensitized lymphocytes upon interaction with a specific antigen [1]. In 1989, Weiser et al. isolated a cDNA encoding human MIF, which allowed further studies on its physiological and pathological characteristics [3]. The identification of MIF’s receptors confirmed its chemokine-like functions and major regulatory abilities in host immune defense and inflammation [4]. According to Human Protein Atlas (HPA) (www.proteinatlas.org), MIF and its partners (e.g., CXCR4, CD74, p53, JAB1) are expressed in all tissues with high levels in bone marrow, reproductive, brain and lymphoid tissues [5–7]. In the Th2 subset of lymphocytes and monocytes/macrophages, in contrast to other cytokines, MIF is present in high intracytoplasmic levels in unstimulated cells. Notably, at least two orders in magnitude lower LPS is needed to stimulate expression of MIF than that in case of TNF-α [8]. In the CNS, MIF is secreted by neurons, Schwann cells, microglia, astrocytes and oligodendrocytes, predominantly in the cortex layer [9, 10]. MIF, a multifaceted cytokine is involved in immune response and numerous biological processes like proliferation, angiogenesis, anti-oxidant signaling and tissue repair [11, 12]. MIF has been identified as a critical controller of inflammation and immune responses by counter-regulating immunosuppressive effect of glucocorticoids [8, 13–15]. Production during systemic inflammation and glucocorticoid regulatory abilities of MIF are in tight conjunction with the hypothalamic–pituitary–adrenal axis. As a pituitary derived stress hormone, MIF became recognized as a key neuroendocrine regulator of immune responses in the CNS [13, 16, 17]. Part of MIF’s pathologic role in inflammatory diseases may be based on neuroendocrine mechanisms [18]. MIF functions in cell proliferation and glucocorticoid action by MAPK (mitogen-activated protein kinases) and cytoplasmic phospholipase A2 activation (cPLA2) [19] (Fig. 1). MIF directly interacts with intracellular protein, JAB1, a coactivator of AP-1 transcription, negatively modulating JAB-1 controlled pathways including cell-cycle regulation [20]. The discovery that MIF is capable of influencing cell proliferation by inactivating p53 tumor suppressor activity may suggest a direct link between inflammation and cancer [21]. MIF exerts its activity via specific receptors including its cognate receptor CD74, and via non-cognate interactions with CXCR2, CXCR4 or CXCR7, or possibly via endocytic engulfment to the cytoplasm [22–24]. MIF binds to the extracellular domain of CD74, initiating CD44 activation and the extracellular signal-regulated kinase-1/2 MAP (ERK1/2 MAP) kinase cascade and downstream inflammatory pathways leading to cell proliferation, cell survival and PGE_2_ production [25] (Fig. 1). Particularly, linkage of MIF with CD74/CD44 regulates the immune response by controlling maintenance, proliferation and survival of macrophages and B cells [26]. Recently, a product of CD74 cleavage, a CD74 cytosolic intracellular domain (CD74-ICD) has been found to induce cell–cell signaling and cell survival in B cells [27]. MIF shares biological functions with its homologue, D-dopachrome tautomerase (D-DT or MIF-2), which in a similar way activates the inflammatory responses [28, 29]. According to HPA, D-DT but not MIF, is greatly produced by white blood cells which suggests that D-DT possesses an especially important role in innate inflammation through CD74 [12]. As for the genetic regulation of MIF expression, there are two promoter polymorphisms located in the *MIF* gene: (1) functional alleles of a − 794 CATT_5–8_ microsatellite repeat that modulates *MIF* mRNA transcription and correlates with MIF expression levels. *MIF* promoter activity is proportional to increased numbers of the CATT repeats at position − 794 [30]: and (2) the nearby − 173 SNP C allele may be associated with increased *MIF* promoter activity by its linkage disequilibrium with the high-expression − 794 CATT_7_ variant.

MIF expression has long been associated with certain diseases such as rheumatoid arthritis, asthma and cancer with increased levels found in more aggressive forms of such diseases [31–34]. In SLE, MIF has been found to play a dual role. High-expression MIF polymorphisms have been found to be associated with a lower incidence of SLE. However, in patients with established disease, low-expression MIF polymorphisms have been linked with a lower incidence of the end-organ injury [35]. Brain disorders from autoimmunity, stroke, cancer and dementia are characterized by an inflammatory component, and MIF has a detrimental effect on these pathologies. In MS, AD and GBM, MIF contributes to the severity of disease [36]. However, its pathological role in brain diseases became challenged based on some recent studies. In Parkinson’s disease (PD), MIF has been found to mediate a neuroprotective effect by suppressing inflammatory responses, inhibiting apoptosis, and inducing autophagy [37]. Moreover, the protective effect of MIF has been reported in amyotrophic lateral sclerosis where elevated MIF levels inhibited the accumulation of misfolded SOD1 [38]. In stroke, cerebral ischemia and depression, MIF has protective as well as pathological roles [36]. From the accumulating data, MIF possesses diverse functions within the CNS and more research is needed to decipher its specific role in normal and pathological conditions.

This review focuses on MIF research and actions in progressive brain diseases such as MS, AD and GBM. As a molecule broadly involved in many biological events and variety of autoimmune or inflammatory conditions, MIF can become a new potential biomarker and therapeutic target for the development of new prognostic, diagnostic as well as treatment strategies. Of note, different approaches to modulating MIF-dependent pathways are now in advanced clinical testing, including neurologic disorders.

## Multiple sclerosis

MS is an autoimmune inflammatory disorder of the CNS characterized by demyelination and permanent neurological disability in young adults with prevalence in women [39, 40]. The most common form of MS at the beginning of disease is relapsing–remitting (RRMS) characterized by spontaneous episodes and partial recovery in disease severity with accumulating neurological dysfunctions over time [39]. After several years, disease mostly progresses into secondary progressive MS (SPMS) with slow permanent advancement of neurological, physical, and mental disfunction [41]. The pathogenesis of MS possesses a strong immune component and MIF as an inflammatory cytokine with potent control over innate and adaptive arms of immunity contributes to the development and progression of the disease [29]. Activation of MIF is essential for regulation of leukocyte migration across the blood–brain barrier [42]. Infiltration of immune cells to the brain tissue causes inflammation, demyelination, and formation of sclerotic plaques, hallmarks of MS.

### MIF profiles in MS patients

MIF drives T cell and macrophage activation and may play a pivotal role in MS. Several different studies have been performed in order to decipher a role of MIF in MS pathogenesis. However, information about MIF expression in MS patients is limited with some contradictions due to variations in groups of MS patients with respect to different stages and severity of disease. In 2000, Niino et al. determined the level of MIF in the cerebrospinal fluid (CSF) of patients with conventional-form multiple sclerosis (C-MS), optic-spinal form multiple sclerosis (OpS-MS), and neuro-Behcet's disease (NBD) [43]. The highest levels of MIF have been found in the CSF of OpS-MS patients in relapse. Elevated levels of MIF were also found in relapsed but not in remission cases of C-MS. In NBD patients, the concentration of MIF in CSF was significantly elevated compared with control samples [43]. Similarly, increased levels of MIF were found in sera of untreated patients with MS relapse indicating their association with MS disease activity [44]. To that point, the high levels of MIF correlated with clinical MRI findings with a worsening EDSS score in different subtypes of MS including clinically isolated syndrome (CIS) [45]. A recent study in CIS patients revealed that observed overexpression of MIF, D-DT, and CD44 appeared to be unique for CD4( +)T cells [46]. In healthy blood MIF is predominantly expressed by B cells [47]. In early MS patients, B cells have been found to have downregulated MIF and MIF receptor (CD74) and upregulated the MIF receptor CXCR4 as compared to healthy controls, potentially reflecting a functional state of anergy that may contribute to the persistence of pathogenic immature B cells in the periphery [47]. In another study, MIF was shown to be highly expressed in human active white matter MS lesions predominantly associated with reactive hypertrophic GFAP + astrocytes and macrophages, suggesting MIF may contribute to the actively demyelinating lesion [48]. A more recent study showed increased levels of MIF both in CSF and in serum of RRMS patients [49]. In contrast, the study by Hjaeresen et al. shows that MIF is decreased during RRMS and increased in SPMS [50]. Additionally, MIF levels were significantly decreased in females with CIS and RRMS as compared to males suggesting sex-dependent regulation of MIF production. These findings are in accordance with our previous study and demonstrate the importance of estrogens and estrogen receptor in inhibition of MIF expression, as well as the binding between MIF and its CD74 receptor in the monocyte sub-population [51, 52]. The findings on how MIF exerts its effect on MS progression in males and females require further clarifications.

### MIF genetic polymorphisms in MS

MIF genetic polymorphism studies support the role of MIF in the pathogenesis and severity of MS. Two polymorphisms in the promoter region of MIF gene have shown correlation with the worsening of autoimmune and inflammatory diseases such as systemic lupus erythematosus (SLE), rheumatoid arthritis (RA) and psoriatic arthritis (PsA) in Mexican-Mestizo population [53–57]. As studied in the white Turkish population, a MIF polymorphism has been associated with younger age of MS disease onset. Patients with multiple sclerosis had an increase in the MIF-173 CC genotype and exhibited significantly lower age of disease onset compared with those with the MIF-173 CG and MIF-173 GG genotypes. The MIF-794 CATT 6/7 genotype had a significantly lower progression index compared with MIF-794 CATT 6/6 and patients with the MIF-794 CATT 5/6 genotype had a significantly later age of disease onset [58]. In another study in the Turkish population, the results suggested no relation between MS susceptibility and MIF gene-173G>C polymorphism [59]. In Mexican patients, the MIF-173 GC genotype was associated with a higher clinical severity of MS [60]. Our study found a correlation between a high expression −794CATT5-8 and associated −173G/C SNP with increased MIF and D-DT levels in males with progressive disease [52]. These findings on the sex-specific contribution of MIF polymorphisms were supported by studies on MS patients in Western Mexico. When grouping by sex, an effect of both MIF polymorphisms (−794 CATT5-8 and − 173 G > C) was found with high MIF serum levels, increased severity and progression in male MS patients [61]. Both studies suggest that MIF polymorphisms could act as sex-specific disease modifiers that increase the severity and progression of MS in male patients. Further confirmation that 173G > C polymorphism can also regulate DDT expression in a sex-specific way and that the DDT is highly expressed in MS brain tissues and promotes MS progression in males but not females has been reported recently [62].

### MIF in the EAE animal model

In experimental autoimmune encephalitis (EAE), an animal model for MS, MIF has been shown to accelerate disease progression, mostly by activation of macrophages and microglia and upregulation of the inflammatory responses in the CNS [48, 63–67]. Moreover, MIF promotes resistance of pathogenic CD4( +) T cells to glucocorticoid treatment in EAE [68]. In SJL mice with severe EAE, treatment with anti-MIF antibodies reduced disease severity and improved recovery by impairment of CNS homing of pathogenic T cells [69]. In another study, MIF-deficient C57BL/6 mice were protected from EAE and treatment with a small-molecule inhibitor of MIF reduced EAE severity in SJL mice [70]. Using MIF-/- mice, it was reported that MIF is necessary for progression of EAE, possibly due to significant decreases in inflammatory cytokines [64]. In our previous study, we demonstrated that MIF or D-DT deficiency ameliorates EAE severity and that D-DT absence is associated with reduced migration of memory and activated mononuclear cells into the CNS. We also showed that genetically controlled high expression of both molecules promotes MS progression in males and that both molecules are important sex-specific disease modifiers [52]. A novel role for MIF in inducing microglial C/EBP-beta, a transcription factor shown to regulate myeloid cell function has also been proposed in a rodent model of MS [48].

### MIF-oriented MS treatments

At present there are no sufficient and satisfactory treatments for MS. The most important caveat in systematically administrated drugs is the limited penetration through BBB. Drugs such as monomethyl fumarate (MMF), a product of dimethyl fumarate (DMF) hydrolysis after absorption inside the small intestine and MTX (mitoxantrone) have only limited access to the CNS. Thus, these drugs would likely have little influence on MIF levels in CNS-resident cells and limited effect on increased MIF levels in CSF as found in RRMS patients [50]. A newer anti-inflammatory drug, ibudilast, a non-selective inhibitor of various cyclic nucleotide phosphodiesterases often used as a bronchodilator for bronchial asthma treatment, plays a central role in processes like inflammation and synaptic plasticity. Ibudilast suppresses pro-inflammatory cytokines, upregulates anti-inflammatory cytokines and blocks TLR4 and acts as a noncompetitive and allosteric inhibitor of MIF tautomerase activity and its chemotactic effects [71]. Additionally, ibudilast possesses an enhanced ability to pass the BBB, and was found in a successful PMS Phase 2 clinical trial to inhibit glial activity, support the production of neurotrophic factors and influence CNS production of MIF [72]. Other therapeutic approaches such as a small molecule inhibitor (ISO-1) and MHC constructs (DRQ) will be discussed below. That said, we are not aware of any studies using MS approved drugs that have evaluated MIF levels.

## Alzheimer disease

Alzheimer disease (AD) is the most common neurodegenerative disease affecting predominantly the hippocampus and cerebral cortex characterized by the aggregation of extracellular Aβ proteins and intracellular neurofibrillary tangles (NFT), which are composed of hyperphosphorylated tau proteins in neurons. Neuroinflammation plays a pivotal role in AD pathogenesis leading to neuronal loss, alterations in glial cells and severe cognitive decline.

### MIF’s effects on glia and neurons in AD

One of the first reports on the MIF’s involvement in AD identified MIF as a new Aβ-binding protein in a soluble fraction of the cerebral cortex of AD brain by immunoprecipitation [73, 74]. Some early reports using immunohistochemistry reported elevated expression of CD74, a MIF receptor in AD [75, 76]. CD74 was found to be increased in microglia in AD cases compared to age-matched controls [76]. Following study revealed a significant increase in CD74 primarily in neurofibrillary tangles, amyloid-beta plaques, microglia and for the first time in neurons of AD cases [75]. Toxic involvement of MIF within amyloid-aggregates was established by studies in brains of transgenic APP mice where MIF has been found to be produced by activated microglia near Aβ plaques [77]. The co-localization of MIF and activated microglia to amyloid deposits has been further confirmed by using mass spectrometry-based imaging technique [78]. Besides microglia, MIF possesses strong influence also on astrocyte activation (Fig. 3). MIF in astrocytes plays an important role in elevated tau phosphorylation, which involves mediators released by the activated astrocytes in AD animal model [79]. In animal models, the expression of MIF in astrocytes and the number of reactive astrocytes were noticeably increased in contrast to MIF knockout mice. Additionally, it has been found that conditioned medium from activated astrocytes could stimulate tau hyperphosphorylation in neurons in a MIF-dependent manner. Recently it has been shown that MIF displays neurotoxicity similar to Aβ [1–42], which was associated with the MIF-induced increase in apoptosis in human neuroblastoma cells [80]. It has been found that MIF’s involvement in neuronal cytotoxicity can be reversed by ISO-1 that blocks the enzymatic and biologic activities of MIF [81]. In vitro data using murine and human neuronal cell lines revealed that ISO-1 almost completely antagonized the neurotoxic effects of Aβ [77]. A recent study on the effect of MIF on inflammatory markers and spatial learning in a mouse model of sporadic AD and on tau pathology in AD patients showed that MIF inhibition resulted in reduced cytokine production in vitro and in vivo [82].

### Sugar metabolic malfunction affects MIFs role in high glucose-induced AD

**Fig. 2:**
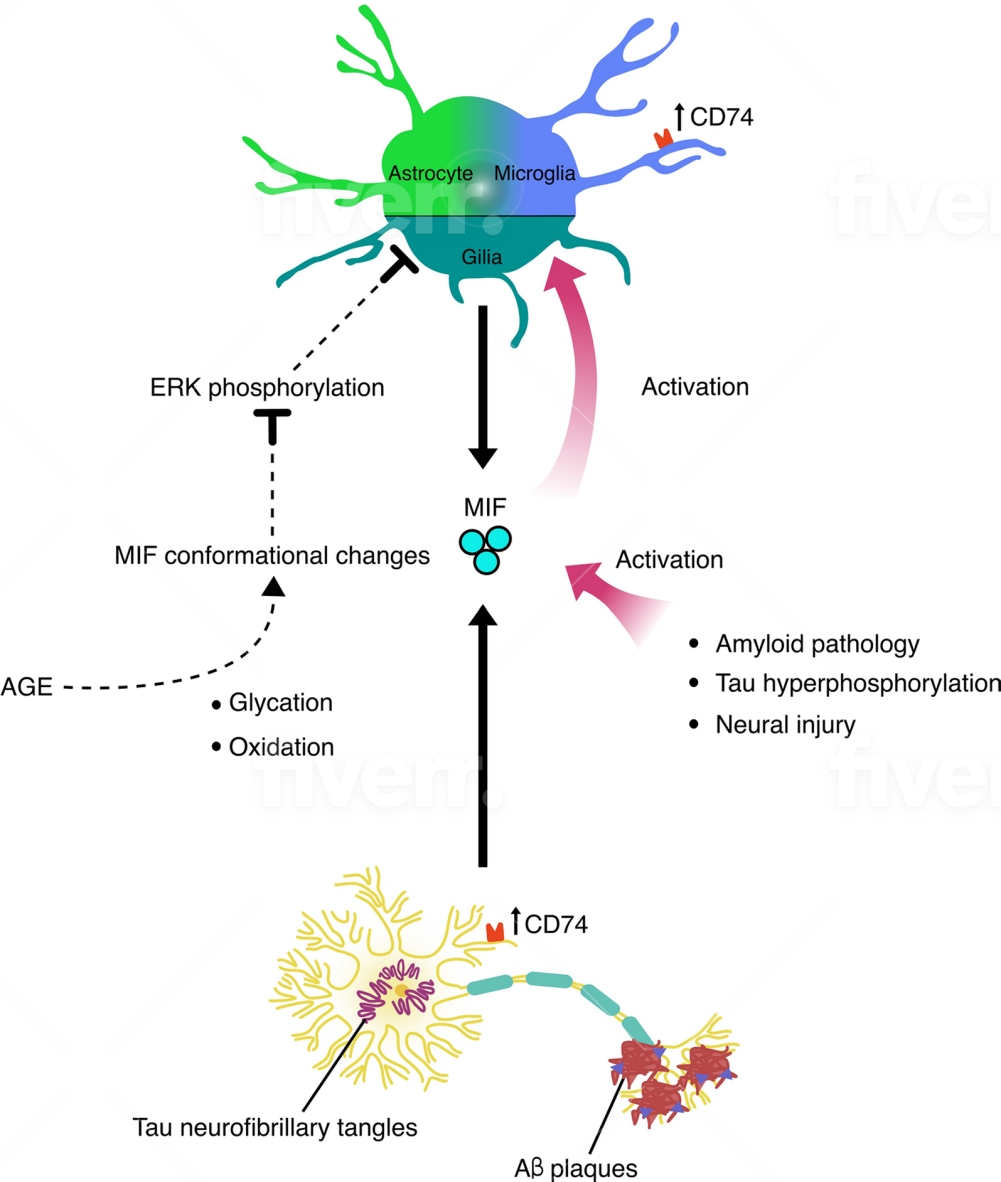
MIF in AD. Activated glia and especially neurons are the main sources of MIF in AD. Dysregulated glucose metabolism and AGE promote MIF conformational changes leading to disrupted ERK signaling in glia cells. In AD increased CD74 has been found in microglia, neurofibrillary tangles, amyloid β plaques and neurons

Some recent studies implicate MIF with progression of high glucose-induced AD. AGEs (advanced glycation endproducts) are neurotoxic, foster the deposition of Aβ and the hyperphosphorylation of tau protein and the expression of proinflammatory mediators in glial cells [83, 84]. It has been demonstrated that AGEs promoted the expression of MIF and aggravated the neuroinflammatory response at the cell level [85]. In PC12 cells, (an AD-cell model), ISO‑1 reduced AGE‑mediated damage by decreasing the expression of neuroinflammatory mediators. Previously, MIF has been found to be glycated and oxidized in AD brain homogenates. The glycation completely inhibited the enzymatic activity of MIF and was harmful to the signaling effects of MIF on glia, strongly weakening MIF-induced ERK phosphorylation [86] (Fig. 2). This might be especially important at the beginning of AD where microglia are actively involved in removing Aβ plaques and MIF signaling is crucial for this beneficial microglia’ function. Thus, dysregulation of glucose homeostasis or insulin regulation leads to MIF conformational changes and severely affects MIF activity with implications for impaired innate immune response during progression of AD [86].

### MIF in AD patients

In human subjects with AD at early clinical stages, cerebrospinal fluid levels of MIF were increased in comparison with age-matched controls, and correlated with biomarkers of tau hyper-phosphorylation and neuronal injury suggesting that MIF can play a role as biomarker for early-stage AD. First report from clinical studies showed significantly increased levels of MIF in the CSF of AD patients in comparison to age matched controls [87]. A possible link between MIF and TNF-α release in AD group is suggested as a correlation between MIF and TNF-*a* concentrations has been found. The next study by this group showed the highest levels of MIF in the brain cytosol and CSF in a mild cognitive impairment group of patients (MCI) that has a high risk to develop AD over time, thus providing evidence that the neuroinflammation occurs early, at predementia stages of AD [77]. A recent study based on measurements of MIF levels in plasma and CSF in MCI or mild dementia (cognitive impairment, CI) patients established a significant role for MIF as biomarker in AD pathology for predicting cognitive failure in MCI and CI [88]. Additionally, this study provided evidence that MIF-related inflammation is related to amyloid pathology, tau hyperphosphorylation, and neuronal injury at the early clinical stages of AD. Further usefulness of MIF as a potential AD biomarker has been proposed by Zhang et al. [89]. In this study, elevated MIF levels were detected in CSF of AD patients but not in MCI or vascular dementia patients. Neurons but not glia cells stimulated with Aβ oligomers were the main source of MIF. Interestingly, reduced MIF expression impaired learning and memory in the AD mouse model thus supporting the conclusion that neuronal secretion of MIF may serve as a defense mechanism to compensate for declining cognitive function in AD. MIF has been found to have neuroprotective abilities on neuronal cells by inducing expression of BDNF, an essential modulator of synaptic plasticity related to learning and memory [90]. MIF administration protected neurons from hypoxic injury by upregulation of mature BDNF and anti-apoptotic molecules in human neuroblastoma cells. Previously, BDNF, serotonin and THP2, a critical enzyme in the biosynthesis of serotonin in the brain have been found to be upregulated by MIF in vitro as well as during both exercise and electroconvulsive seizure in vivo [91].

## Glioblastoma

Glioblastoma (GBM) is a grade IV astrocytoma derived from astrocytes according to WHO classification [92]. GBM is the most common and the most deadly brain tumor with low treatment efficacy after surgery, chemotherapy and radiation. One of the main reasons for poor therapeutic outcome in this type of cancer is marked cellular heterogeneity with genetic and epigenetic variability [93]. Recent genome-wide association studies (GWAS) showed that genetic susceptibility to GBM and non-GBM tumors are highly distinct with possible different etiologies [94].

### Pro-carcinogenic abilities of MIF

**Fig. 3:**
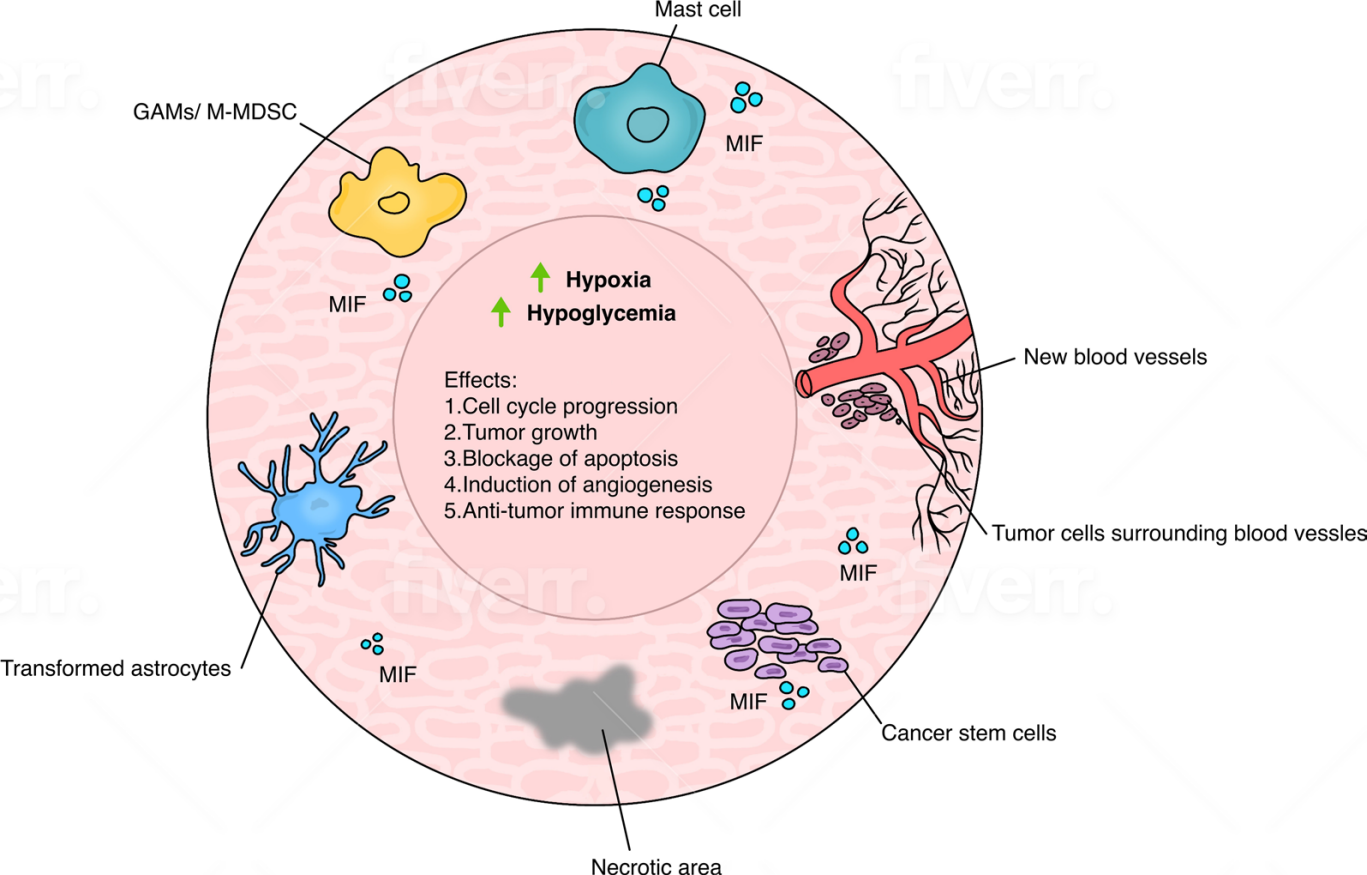
Main MIF sources and its effects in GBM. One of the key stressors in development of GBM are hypoxia and hypoglycemic states which induce production of high MIF levels in primary GBM cells leading to neovascularization. MIF major sources are: 1. cancer stem cells (CSC) located nearby new blood vessels; 2. transformed astrocytes and 3. MCs. All sources add to promotion of immune suppression mostly by MDSCs, angiogenesis and increased cell proliferation

Despite MIF’s critical role in the pathogenesis of inflammatory and immune responses, it has been documented that MIF promotes carcinogenesis and its elevated levels have been found in various tumors [95–97]. MIF is able to promote cellular processes related to tumorigenesis such as cell cycle progression, tumor growth, blockage of apoptosis, induction of angiogenesis and tumor spread [98] (Fig. 3). Moreover, MIF has profound and detrimental effects on anti-cancer immune responses. This includes inhibition of NK anti-cancer cytotoxic effect, inhibition of T cell activation, promotion of MDSCs (myeloid-derived suppressor cells) and polarization of macrophages towards an anti-inflammatory phenotype [99–103]. Blockade of MIF by shRNA in glioma cells restores cytotoxic activity of NK and CD8 + T cells downregulating the immune receptor NKG2D [104]. In contrast to abundant studies showing that MIF is a key factor in tumor immune response, recently it has been found that its cognate receptor CD74 is confined to human microglia/macrophages and is positively associated with pro-inflammatory anti-tumor immune responses and improved patients’ survival [105]. Considering the extremely high diversity of microglia subpopulations with unique gene expression profiles and different roles, more studies are needed to decipher the role of CD74 in microglia anti-tumor responses.

High levels of MIF predict poor survival rates in cancer patients [106, 107]. In prostate cancer and in many solid tumors, increased risk is associated with polymorphism in the C allele in the MIF-173 G/C promoter single-nucleotide polymorphism (SNP) as documented by meta-analysis. MIF gene promoter polymorphisms also are associated with lymphatic metastasis and cervical cancer [108, 109]. MIF is highly expressed by GBM cells and higher levels of MIF are associated with increased cancer grade [104, 110] as well as poor prognosis and tumor recurrence [103, 111]. One of the critical stressors in the development of GBM are hypoxia and hypoglycemic states which induce production of high MIF levels in primary GBM cells leading to neovascularization [110, 112] (Fig. 3). Hypoxia-induced MIF expression is driven by HIF-1, but amplified by hypoxia-induced degradation of cAMP-responsive element binding protein (CREB) [113]. In the hypoxic area of glioma specimens, MIF co-localized with CXCR4 where MIF promotes vasculogenic mimicry formation [114].

### MIF effects on the cellular environment in glioma

In the CNS, MIF is produced by neurons, astrocytes, oligodendrocytes, microglia and Schwann cells [10, 115]. Activated microglia and macrophages play a central role in the delivery of MIF and signaling molecules to nearby neurons and transformed astrocytes [116, 117]. An increased number of activated microglia that reside in the tumor periphery and infiltrating macrophages that occupy the perivascular area account up to 30–50% of all cell types in gliomas and correlate with higher grade and worse prognosis [118]. The number of glioma-associated microglia/macrophages (GAMs) and MDSCs is the highest in malignant gliomas and inversely correlates with patient survival [103, 117]. MDSCs have been identified nearby cancer stem cells (CSC) which are responsible for high MIF production and promotion of immune suppression activities of MDSCs [119]. Depletion of MDSCs by 5-fluorouracil (5-FU) increases the survival rate in mice with glioma [119]. In vitro treatment with sulforaphane, a MIF inhibitor, reverses the transition of normal monocytes to MDSCs [120]. Higher numbers of the circulating monocytic subset of MDSCs (M-MDSC), in contrast to macrophages and regulatory T cells, have been associated with higher tumor grade and poorer prognosis in GBM [103, 121]. Recently it has been found that in both murine and human models, M-MDSCs express high levels of the CD74 MIF receptor and are localized in the glioma microenvironment, in contrast to granulocyte-MDSCs (G-MDSCs) which show minimal accumulation in the tumor environment [103]. Ibudilast which appears effective in an experimental model of glioma, suppresses MDSCs and boosts activity of CD8 T cells [103]. Additionally, mast cells (MCs) have a crucial role in glioma pathogenesis that contribute to angiogenic processes and immune regulation [122]. MIF attracts mast cells to the tumor environment and accumulation of MCs and their pSTAT5 expression correlates with the level of MIF [123]. MIF expression was frequently associated with the presence of the tumor-suppressor gene p53, which supports stem cell tumorigenic activities [110]. Many stem cells, including human pluripotent stem cells (iPCS), were the source of MIF [124]. Blockade of MIF by miR-608 in glioma stem cells reduced the proliferation, translocation, and invasion [125]. MIF gene knockdown effected brain tumor initiating cell (BTIC) function through direct, intracellular inhibition of p53, reducing cell proliferation and increasing apoptosis in an in vitro setting and in human mouse xenograft model [126].

### MIF and D-DT signaling in cancer

MIF signals though several different receptors primarily via cognate CD74 and non-cognate receptors CXCR2, CXCR4 and CXCR7 [4, 25, 127, 128]. Upon binding to CD74 in combination with the CD44 co-receptor, the signal is transduced via src/MAPK signaling pathway, whereas binding to CXCR2, MIF signaling is PI3K/Akt-dependent [128]. MIF activates MAPK and PI3K pathways involved in signal transduction cascades in many cancers [103, 121, 129–131]. Additionally, the PI3K pathway contributes to the MIF-mediated suppression of apoptosis linked with p53 activation [132]. It has been found that in melanoma, CD74-MIF interaction promotes tumor survival via the PI3K/AKT pathway in response to IFN-γ [133]. A second ligand for CD74 is D-DT (*a.k.a*. MIF-2) with close structural and functional similarities with MIF and an overlapping spectrum of activities such as tumor formation, growth and survival of cancer cells and tumor invasion [134, 135]. Both molecules cooperate in tumorigenesis. For example, in non-small cell lung carcinoma both molecules via CD74 negatively regulated AMP-activated protein kinase (AMPK) leading to increased expression of CXCL8 and VEGF [136, 137]. In pancreatic cancer tissue, D-DT was over-expressed together with MIF and knockdown of D-DT and MIF in a pancreatic cell line, PANC-1, cooperatively inhibited ERK1/2 and AKT phosphorylation, increased p53 expression, and reduced cell proliferation, invasion and tumor formation. A covalent tautomerase inhibitor of both DDT and MIF, a 4-iodo-6-phenylpyrimidine (4-IPP), attenuated cell proliferation and colony formation in vitro and tumor growth in vivo [138]. 4-IPP irreversibly binds to Pro1 of MIF or D-DT via nucleophilic dislocation by Pro1 of an aromatic iodo group and was shown in vitro to have anticancer properties in the head and neck squamous cell carcinoma cell line, SCCVII, and in the human A549 lung adenocarcinoma [139]. In GBM, targeted inhibition of MIF and D-DT by 4-IPP might improve radiation therapy [140]. Additive effects have been shown in dual inhibition of MIF and D-DT by shRNA treatment in clear cell renal cell carcinomas [141]. Current treatment strategies are focused on simultaneous counteracting of both cytokines.

### Anti-MIF treatment strategies in glioma models

Several different anti-cancer and GBM treatment approaches based on MIF inhibition have been proposed and include competitive, irreversible and endogenous inhibitors, molecules that destabilize MIF, and monoclonal antibodies blocking MIF or CD74 [34, 103, 142]. One of the biggest caveats in glioma treatment strategies is the inability of drugs to traverse the blood–brain barrier (BBB). Attempts have been made to design more lipophilic compounds with better ability to reach CNS tumors. Recently this direction of research has become focused on nanotechnology [143, 144]. So far, only liposomes have reached phase I/II clinical trials [143]. One of the well-known MIF competitive inhibitors is ISO-1. ISO-1 reduces the proliferation of human glioblastoma cell lines, especially the human LN18 cell line, in a dose-dependent manner and was able to restore contact inhibition, reduce proliferation and mitogenic signaling [112, 145]. Moreover, ISO-1 was able to sensitize glioma cells to glucocorticoids, and when applied together with dexamethasone, cell migration and invasion were diminished in Hs683 glioma cells [146]. MIF knockdown by antisense transfection allowed for restoration of contact inhibition in human glioblastoma cell lines [145]. Blockade of MIF with shRNA resulted in an increase of CD8-positive CTLs and reduction of Treg lymphocytes in the brain in animal models of glioma [119]. Silencing of CD74 by shRNA was associated with reduced AKT and ERK1/2 pathways and in the human glioma U87 cell line, significantly suppressed proliferation and increased temozolomide sensitivity [147]. Monoclonal antibodies against MIF have been tested in in vitro settings where they were able to reduce growth of glioma cell lines, the migration of cells and arginase-1 assembly in MDSCs in a CXCR2-dependent manner [119, 123, 145]. Treatment with 4-IPP (inhibitor of MIF or D-DT) showed the potential to improve radiotherapy by inhibiting the stemness and intracellular signaling pathways and inducing apoptosis in vitro and in vivo glioma models [140]. A common chemotherapy in glioma can be efficiently enhanced by using combined treatments. Synergism in the inhibition of cell cycle and increased apoptosis has been observed in ex vivo and in vivo models when ibudilast was combined with temozolomide leading to significant increased overall survival [148]. Despite some successful results in the in vitro experiments mentioned, more studies evaluating molecules and their receptors with known genetic polymorphisms are needed to help establish the clinical relevance of potential therapeutics in GBM.

## Discussion of MIF and CD74 inhibitors for possible clinical use in MS, AD and astrocytomas

### MIF-inhibitors as novel neurotherapeutics

Once CD74 was determined to be the cognate receptor for MIF [25], additional CD74 targeted pharmacologic approaches became available for consideration in CNS disease. A monoclonal antibody targeting CD74 (milatuzumab) has received US FDA approval for multiple myeloma [149], a malignancy of B cells where MIF–CD74 survival pathways are highly active [150]. Milatuzumab further showed evidence of efficacy in lupus nephritis, a highly inflammatory condition, in one phase 1B study [151]. Anti-MIF (imalumab) completed phase II testing in heavily pre-treated cancer patients with a favorable safety profile [152], but has yet to be evaluated further. The rational design of small-molecule antagonists of MIF/CD74 interaction has been facilitated by MIF’s vestigial tautomerase active site, which overlaps structurally with the CD74 binding domain [153, 154]. This feature has been exploited by several academic and industry groups to design small-molecule MIF tautomerase inhibitors that target this site [155–158] and a subset of such inhibitors shows therapeutic activity in mouse models of autoimmunity [159, 160]. The small molecule MIF antagonist that is furthest advanced in clinical development is ibudilast, which was originally developed as a phosphodiesterase inhibitor but was discovered to inhibit MIF allosterically [71]. Remarkably, ibudilast binds to a dynamic site that is not present in the (apo) crystal form of MIF; that is, this site is only revealed when ibudilast binds to MIF. Once bound, the ensuing conformational changes eliminate MIF activity. Ibudilast has shown efficacy in a phase II study of MS, where high-expression *MIF* genotype is a risk for progressive disease [52, 161]. Ibudilast is used for asthma in Japan and is in clinical testing in the US for additional inflammatory conditions, as well as in oncology and in neurodegenerative disease conditions.

The recent appreciation that the MIF family member D-DT is co-expressed with MIF in many disease conditions, and also activates CD74 has prompted interest in dual MIF/D-DT inhibitors [162, 163], and possibilities exist for targeting both MIF and D-DT together by either small molecule or bispecific antibody approaches [164, 165].

### Major histocompatibility complex (pMHC) constructs represent a novel therapeutic approach for treatment of PMS and other conditions involving activation of the CD74 pathway

*RTL1000:* In the mid-1990s, our lab designed recombinant T cell receptor ligands (RTLs) that mimic the major histocompatibility complex (MHC) class II/peptide interface with cognate T cell receptors [166–168]. The initial RTL1000 construct, designed to partially mimic only the extracellular components of MOG-35-55 specific T cell receptor (TCR) ligands present on antigen presenting cells, consisted of the alpha-1 and beta-1 domains of HLA-DR2 linked to the MOG-35-55 peptide. Interaction of the soluble RTL1000 with the TCR of MOG-35-55 specific CD4 + T cells produced partial, but incomplete, T cell activation that selectively blocked T cell proliferation and IL-2 secretion of MOG-35-55 specific T cells. In a series of studies, we demonstrated that partial major histocompatibility complex (pMHC) constructs bind to human and mouse monocytes through cell surface CD74 and that this interaction inhibits MIF binding and signaling [65, 169]. The RTL1000 construct could strongly inhibit EAE, a mouse model of MS in HLA-DR2*1501 transgenic mice [170]. This model was relevant to ~ 50% of MS patients that were DR2 positive. RTL1000 was approved by FDA for a Phase 1 clinical trial carried out in 2007–2009 which demonstrated safety and tolerability of RTL1000 at a ≤ 60 mg dose given i.v. [171, 172].

*DRα1-MOG-35–55:* We later learned that another major receptor for RTL1000 (besides the TCR) was the highly conserved CD74 molecule [169], which also was the receptor for MIF and D-DT. By slightly altering the RTL1000 design (removal of the DR2 beta 1 domain that contained histocompatibility markers), we produced a simplified ligand, DRα1-MOG-35-55, for CD74 that could competitively inhibit MIF/D-DT signaling, block downstream inflammatory activity (ERK-1/2 phosphorylation and MAPK activation) and treat EAE in both DR2*1501 transgenic and wild-type C57BL/6 mice comparably with RTL1000 that was active only in the DR2 transgenic mice [66, 166]. We found that the DRα1-MOG-35-55 construct displayed a stronger effect in downregulating CD74 expression on male CD11b + cells as compared to female cells and the treatment of chronic EAE in female mice was dependent on signaling through ER-α [51], a restriction that we could overcome with higher doses of DRα1-MOG-35-55. Due to conservation of DRα1 in humans and mice and molecular modeling implicating DRα1 but not DRβ1 amino acid residues as the major binding region for CD74, the DRα1-MOG-35-55 construct was confirmed to retain ability to block CD74 signaling and reverse clinical signs of EAE independent of MHC barriers [173]. The DRα1 construct can inhibit the activation and recruitment of brain-infiltrating T cells and CD11b + CD45hi myeloid cells and expression of the co-stimulatory CD86 marker on CD11b + CD45hi cells, and at the same time increase expression of the anti-inflammatory CD206 marker on CD11b + CD45int microglial cells [173]. Moreover, DRα1-MOG35-55 treatment could significantly reduce severe disease enhancing effects of MIF and D-DT in both male and female wild-type and knockout mice with chronic EAE [174, 175].

**Fig. 4:**
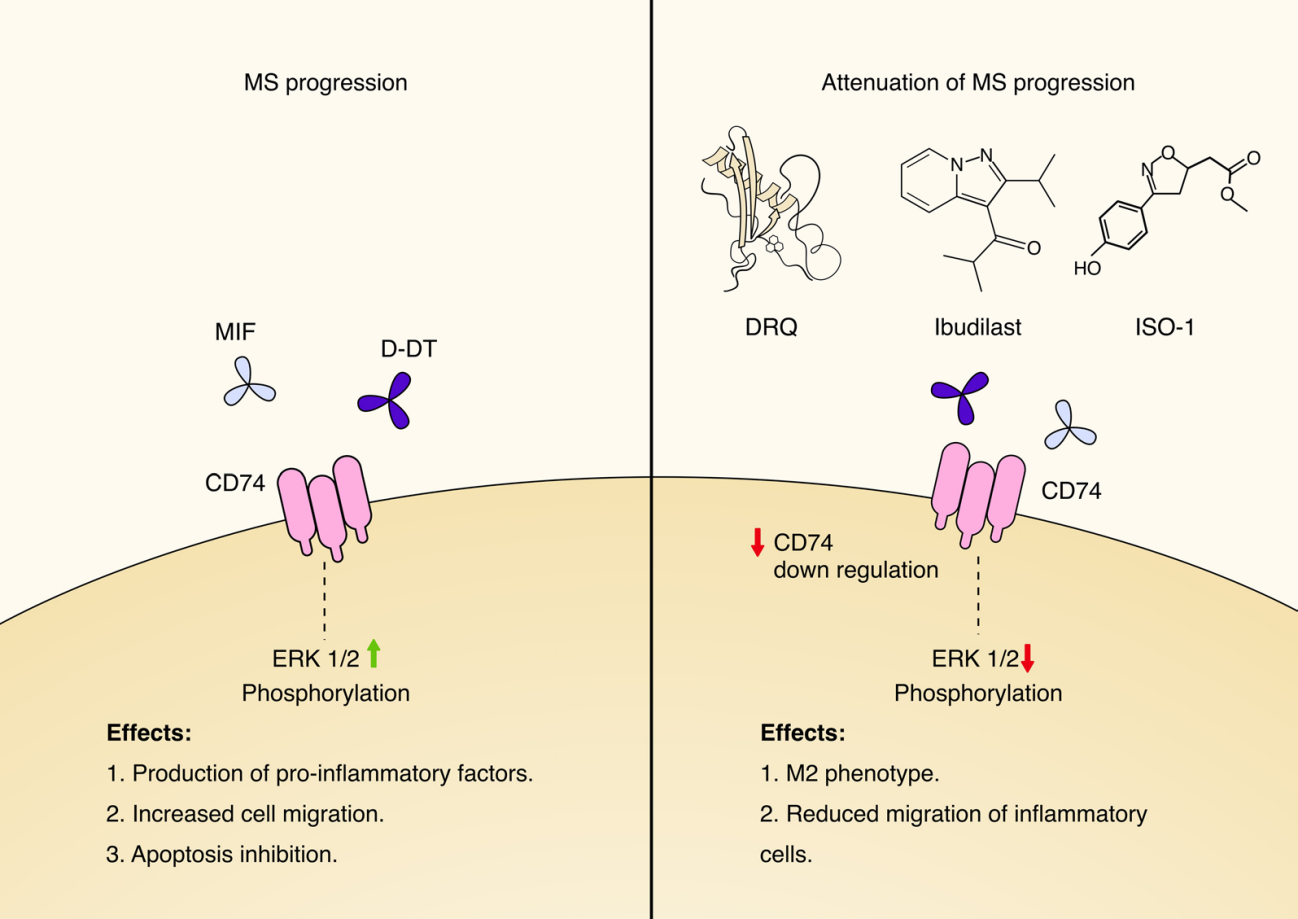
MIF and D-DT inhibition in MS. MIF and D-DT signaling through CD74 is involved in MS progression by increasing inflammatory cell migration to the CNS, enhancing secretion of pro-inflammatory cytokines and prolonging survival of pro-inflammatory cells. Inhibition of MIF or D-DT signaling by partial MHC constructs (DRQ), ISO-1 or ibudilast attenuates signs of MS progression

*DRQ:* DRQ is a third generation 13.5 kDa protein construct comprising the human (h)MOG-35-55 peptide covalently linked to the L50Q modified DR alpha-1 domain of MHC class II [176]. This modified construct had eightfold higher inhibitory activity for blocking MIF/D-DT binding and downstream signaling through CD74 and enhanced ability to reverse EAE induced paralysis in wild-type mice. DRQ is thus our prime candidate for treatment of progressive MS as well other MIF/CD74-dependent inflammatory conditions, including stroke, methamphetamine abuse, traumatic brain injury, Alzheimer’s disease and GBM without the need for histocompatibility testing of DRQ recipients [173]. Inhibition of MIF and/or D-DT signaling by DRQ, ISO-1 or ibudilast may have the potential to slow MS progression (Fig. 4).

## Concluding remarks

MIF is a pleiotropic protein that functions as a glucocorticoid-induced immunoregulator, pituitary hormone, inflammatory cytokine, and immune and growth response regulator, with pathologic roles in autoimmunity, neurologic disorders, and oncology. There is a growing interest in the therapeutic application of MIF antagonists in different diseases. Increased studies of D-DT, a MIF homolog molecule with overlapping biological functions and possible synergism as MIF, has focused attention on the utility of dual inhibition for the full potential of therapy. Possible pharmacological strategies include small molecules that disrupt both MIF and D-DT interaction with CD74, either as competitive or irreversible inhibitors. In this regard, 4-IPP, is one dually active, irreversible inhibitor. Anti-CD74 similarly prevents both MIF and D-DT signaling, and the bio-engineering of dual specificity antibodies (e.g., binding to both MIF and D-Dt) can be envisioned. A novel therapeutic approach utilizing our DRQ construct to inhibit dual effects of MIF and D-DT signaling has the potential to treat all progressive brain diseases involving CD74-dependent neuroinflammatory pathways. In Table 1, we indicate the operative mechanisms of action of MIF and D-DT in MS, Alzheimer disease and glioblastoma and potential therapeutic anti-MIF/D-DT drugs that could block their pathogenic effects. Additionally, the development of new technologies that identify genetic heterogeneity of cellular subpopulations responsible for pathology such as single cell analysis, cellular bar coding, CRISPR-Cas 9 and CyTOF hold future promise for new therapeutics. Finally, the circumstance that approximately 20% of individuals express a high expression *MIF* allele [52] supports the possibility that MIF/CD74 directed therapies would be most effectively used in such subjects, thus providing a more precise pharmacogenomic for treatment of a number of MIF-dependent illnesses. Further studies are needed to decipher how MIF inhibitors block the hyperactivation of cells, including glia cells in the CNS, and exert anti‑inflammatory and neuroprotective effects.

**Table 1:** The operative mechanisms of action of MIF and D-DT in MS, Alzheimer disease and glioblastoma and potential therapeutic anti-MIF/D-DT drugs that could block their pathogenic effects

Disease	MIF mechanism of action	MIF targeting
Multiple sclerosis	• Induces leukocyte migration [52, 69] • Activation of macrophages, astrocytes and microglia [48, 63–67] • Promotes resistance of CD4( +) T cells to glucocorticoid treatment [68] • Increases pro-inflammatory cytokine secretion [52, 64]	• Partial MHC class II constructs [66–69, 169–176] • Ibudilast [52, 72, 161] • ISO-1 [70]
Alzheimer disease	• Secreted by microglia near Aβ plaques [77, 78] • Activation of astrocytes [79] • Induces neurotoxicity [77, 85]	• ISO-1 [81, 112, 145, 146] • Sulforaphane [120] • Ibudilast [103, 148] • mIR-608 [125] • 4-IPP [140]
Glioblastoma	• Induces cell cycle progression, tumor growth, blockage of apoptosis [108, 109, 131] • Induces angiogenesis and tumor spread [98] • Inhibition of NK and T cell anti-cancer cytotoxicity [99–103] • Promotes MDSCs and M2 macrophage polarization [99–103]	• ISO-1 [112, 145] • shRNA MIF inhibitor [119] • Monoclonal antibodies against MIF [118, 123, 145]

## Data Availability

Not applicable.
